# Performance of Large Language Models in Patient Complaint Resolution: Web-Based Cross-Sectional Survey

**DOI:** 10.2196/56413

**Published:** 2024-08-09

**Authors:** Lorraine Pei Xian Yong, Joshua Yi Min Tung, Zi Yao Lee, Win Sen Kuan, Mui Teng Chua

**Affiliations:** 1 Emergency Medicine Department National University Hospital National University Health System Singapore Singapore; 2 Department of Surgery Yong Loo Lin School of Medicine National University of Singapore Singapore Singapore; 3 Urgent Care Centre Alexandra Hospital National University Health System Singapore Singapore; 4 Department of Urology Singapore General Hospital Singapore Singapore

**Keywords:** ChatGPT, large language models, artificial intelligence, patient complaint, health care complaint, empathy, efficiency, patient satisfaction, resource allocation

## Abstract

**Background:**

Patient complaints are a perennial challenge faced by health care institutions globally, requiring extensive time and effort from health care workers. Despite these efforts, patient dissatisfaction remains high. Recent studies on the use of large language models (LLMs) such as the GPT models developed by OpenAI in the health care sector have shown great promise, with the ability to provide more detailed and empathetic responses as compared to physicians. LLMs could potentially be used in responding to patient complaints to improve patient satisfaction and complaint response time.

**Objective:**

This study aims to evaluate the performance of LLMs in addressing patient complaints received by a tertiary health care institution, with the goal of enhancing patient satisfaction.

**Methods:**

Anonymized patient complaint emails and associated responses from the patient relations department were obtained. ChatGPT-4.0 (OpenAI, Inc) was provided with the same complaint email and tasked to generate a response. The complaints and the respective responses were uploaded onto a web-based questionnaire. Respondents were asked to rate both responses on a 10-point Likert scale for 4 items: appropriateness, completeness, empathy, and satisfaction. Participants were also asked to choose a preferred response at the end of each scenario.

**Results:**

There was a total of 188 respondents, of which 115 (61.2%) were health care workers. A majority of the respondents, including both health care and non–health care workers, preferred replies from ChatGPT (n=164, 87.2% to n=183, 97.3%). GPT-4.0 responses were rated higher in all 4 assessed items with all median scores of 8 (IQR 7-9) compared to human responses (appropriateness 5, IQR 3-7; empathy 4, IQR 3-6; quality 5, IQR 3-6; satisfaction 5, IQR 3-6; *P*<.001) and had higher average word counts as compared to human responses (238 vs 76 words). Regression analyses showed that a higher word count was a statistically significant predictor of higher score in all 4 items, with every 1-word increment resulting in an increase in scores of between 0.015 and 0.019 (all *P*<.001). However, on subgroup analysis by authorship, this only held true for responses written by patient relations department staff and not those generated by ChatGPT which received consistently high scores irrespective of response length.

**Conclusions:**

This study provides significant evidence supporting the effectiveness of LLMs in resolution of patient complaints. ChatGPT demonstrated superiority in terms of response appropriateness, empathy, quality, and overall satisfaction when compared against actual human responses to patient complaints. Future research can be done to measure the degree of improvement that artificial intelligence generated responses can bring in terms of time savings, cost-effectiveness, patient satisfaction, and stress reduction for the health care system.

## Introduction

Patient complaints often arise from perceived breaches in expected standards of care and are common in health care institutions globally. In the UK’s National Health Service alone, over 100,000 such grievances were lodged annually [1], a number that has been on the rise in recent years [2]. To address these complaints, hospitals typically engage the complainant in a dialogue, with the objective of investigating potential problems in health care delivery or experience, and to reach a resolution that may involve an apology, rejection, or compensation. This process often involves much time and effort on the part of the health care workers, with 1 study suggesting as much as 3326 person-hours a year spent managing these complaints [3].

Despite significant advancements in complaint handling systems across various countries, such as the Netherlands’ Client Right of Complaint Act and the UK Parliamentary and Health Service Ombudsman’s complaints handling framework, dissatisfaction remains high [4]. Prior investigation has shown that up to 61% of patients expressed dissatisfaction with complaint handling by the time of closure of the complaint file [5], and one-third of complainants remained unhappy even when their complaints had been deemed valid and formally put in the right [6].

The goals of restoring patients’ satisfaction and confidence in health care institutions are often not achieved through traditional complaint resolution methods [5,7,8]. Large language models (LLMs) such as ChatGPT, which are trained on a vast corpus of text data, offer a potential alternative approach. These artificial intelligence (AI) models have recently been shown to hold promise in numerous use-cases in the health care setting, including patient communications [9-11]. Ayers et al [12] also demonstrated that responses from ChatGPT to patient questions were rated as more detailed and more empathetic than responses from physicians [12]. Hence, they can potentially improve efficiency and effectiveness in replying to patient complaints, thereby reducing time required and rechanneling resources to other aspects of the health care system.

The objective of this study was to evaluate the performance of LLMs in addressing patient complaints, with the goal of enhancing patient satisfaction and trust in health care systems.

## Methods

### Complaint Response Generation

Deidentified patient complaint emails from August 2022 to February 2023, and their associated responses, were obtained from the patient relations department (PRD) of National University Hospital, Singapore. The source of the complaints came from various departments, including outpatient clinics, emergency department and inpatient wards. These patient complaint scenarios were categorized using the Healthcare Complaints Analysis Tool [13] to ensure a mix of different types of patient complaints being evaluated. They were first sorted into 3 main domains of clinical, management, and relationship problems then into 7 main problem categories: quality, safety, environment, institutional processes, listening, communication, and respect and patient rights. Each patient complaint scenario was further subcategorized within the main problem categories to classify the specific types of problems within each complaint (eg, problems in institutional processes were subcategorized into problems in bureaucracy, waiting times, or accessing care).

ChatGPT-4.0 (OpenAI, Inc) with an 8000-token context length was provided with the complaint emails in August 2023 and prompted to generate appropriate responses. ChatGPT was instructed to assume the role of a patient relations manager with the prompt as shown in Textbox 1. Prompts for style included word-limit restrictions to 250 or 350 words depending on the complexity of the complaint, due to ChatGPT’s known tendency toward verbosity [14,15]. Responses were generated for a total of 19 patient complaints.

**Prompt provided to ChatGPT.**

Please assume the role of patient relations manager in a busy public hospital and provide a reply to the following email sent in by members of the public. Please also limit the reply to a maximum of 250 words unless the case is complex and you are unable to address all issues with the default word count, in which case a higher word count limit of 350 words is permissible.

### Ethical Considerations

This project was exempted from formal review by the National Healthcare Group Domain Specific Review Board, Singapore as it was a quality assessment project with no additional risks beyond usual clinical practice (reference number 2023/00327). Furthermore, our data did not contain any identifiers and the study did not involve direct patient care.

### Comparison of Responses

Over a period of 1 month in September 2023, participants were randomly selected and invited to participate on a voluntary basis. They were asked to complete an anonymized web-based questionnaire administered via Google Forms (Google LLC), comparing responses generated by ChatGPT, against actual human responses created by patient relations officers in the PRD. Google Forms was selected as the survey platform for ease of access by the respondents to enhance completion rate. Demographic data including age, gender, and occupation (health care worker vs non–health care worker) were collected. Health care workers comprise anyone who works in a health care or community care setting, including management and support staff whose roles may not involve direct patient contact. Responses were scored on four 10-point Likert scales for (1) appropriateness and completeness, (2) empathy, (3) satisfaction, and (4) an overall ranking item. The 10-point Likert scale ranged from 1=very poor, not empathetic, very dissatisfied to 10=very good, very empathetic, very satisfied. A 10-point Likert scale without a midpoint was chosen to avoid a neutral stand and allow an unequivocal selection [16]. Participants were also asked to assume the role of a patient and to choose a preferred response (response A or response B) for each complaint. Responses generated by humans and ChatGPT were randomized to A or B for each scenario and participants were blinded to this. An example of a survey scenario and its corresponding responses are shown in Table 1.

**Table 1:** Example of a complaint scenario.

Domain, category, and subcategory	Scenario	ChatGPT response	Human response
Relationship problems, respect and patient rights, and disrespect	“I would like to feedback on my recent visit to the XXX clinic around noon. Usually the front line service counter staff are very pleasant, special mention to staff AAA and another guy. However, this visit they weren’t around. I would like to feedback on the very unpleasant experience with staff BBB (saw the counter name tag), her personal name tag on uniform is not visible. I merely approached her and asked her a question when she wasn’t serving anyone, but she gave a death stare and has a very rude attitude in terms of voice and tone. It is really uncalled for such hostile behavior from a front line personnel. This hospital usually has high standards and excellent Customer service. I think this matter needs attention and to be addressed as many other patients may have to endure such undeserving attitude when the hospital environment ought to be warm and kind. Please follow up on this and hear from you soon. Thank you.”	“Dear [Name], Thank you for taking the time to share your feedback regarding your recent visit to our XXX clinic. I'm pleased to inform you that, upon receiving your feedback, we immediately brought the matter to the attention of the Clinic Manager. Staff BBB has since been counselled regarding her behavior, and we want to assure you that we are taking active steps to address this issue. Her performance and interactions will be monitored closely and will be a significant aspect of her annual performance review. We sincerely regret the experience you had and understand the importance of maintaining a welcoming and supportive atmosphere at our clinics. It's crucial for patients to feel comfortable and valued during their visits. I'm also grateful for your commendation of Staff AAA and other frontline staff. It is our constant endeavor to ensure our patients receive the best care and service. Your feedback is pivotal in our mission to uphold and even surpass the high standards we set for ourselves at this hospital. Please know that your voice has initiated change, and we hope that future visits will reaffirm your faith in our commitment to excellent patient care. Thank you for helping us improve, and please do not hesitate to reach out with further concerns or feedback. Warm regards.”	“Dear [Name] Thank you for your email. We are sorry for your experience. Please be informed that the clinic manager has counselled the said staff. In addition, we have taken the opportunity to reiterate to the team on the importance of providing good service to patients and visitors. The staff’s performance will be monitored closely and reflected in their yearly review. Once again, thank you for writing to us. It is with feedback like yours that helps us improve our services. Please feel free to contact us again if you need any assistance. Thank you.”

### Sample Size

We initially targeted 20 scenarios for this study, based on a conservative estimate of 70% of our study participants preferring ChatGPT compared to human responses [12], which would provide 95% confidence level and at a preset α of 5%. However, a total of 19 scenarios were eventually included for the survey due to availability of suitable scenarios; this yielded 92% confidence at a preset α of 5%. A power of 92% would still be within the conventional β value of .05 to .20 (ie, a power of 80% to 95%) [17]. We achieved 188 complete responses which gave an estimated margin of error of 7% and 95% confidence level [18].

### Statistical Analyses

Statistical analyses were conducted using Stata 17 (StataCorp) and SPSS Statistics (version 26; IBM Corp). For categorical variables, chi-square or Fisher exact tests were used for analysis as appropriate; nonparametric variables are reported as medians with their IQRs and analyzed with Wilcoxon signed rank test (for matched groups) or Mann-Whitney *U* test. We additionally compared if there were differences in responses between male and female, and health care versus non–health care workers. Linear regression analyses were performed to determine if word count of responses impacted scores in each of the 4 questionnaire items (appropriateness, empathy, quality, and satisfaction). A *P* value of <.05 was considered statistically significant.

To investigate if the effect of authorship on scores was mediated by word count, a simple mediation analysis was performed post hoc using PROCESS v4.2 macro for SPSS [19]. The outcome variable analysis was each of the 4 item scores. The predictor variable was authorship and mediator variable for the analysis was word count. Indirect effects of word count on item scores were considered statistically significant if the 95% CI did not cross 0.

## Results

During our study period, there were 188 participants whose age ranged from 19 to 70 years, with median age of 37 (IQR 32-42) years. There were almost equal proportions of male (n=89, 47.3%) and female (n=97, 51.6%) participants, and predominantly health care workers (n=115, 61.2%). Responses generated by ChatGPT averaged 238 words in length, in comparison to an average of 76 words for responses written by PRD staff. A statistically significant difference in scores across all questionnaire items was noted, in favor of ChatGPT (Table 2).

**Figure 1:**
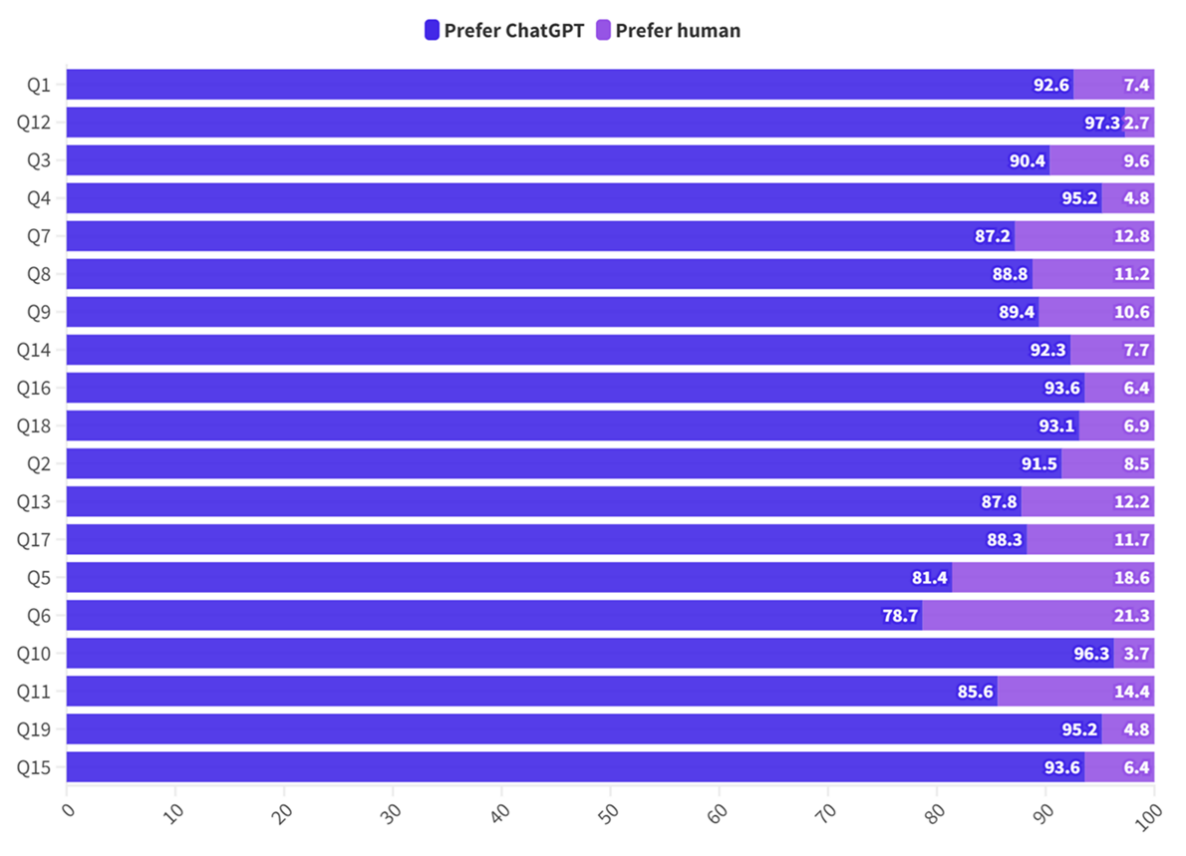
Preference for ChatGPT versus human response.

The majority of respondents preferred the replies provided by ChatGPT across all domains of management, clinical and relationship problems, ranging from a proportion of 87.2% (n=164) to 97.3% (n=183) of participants (Tables 3 and 4, and Figure 1). A large proportion of the median scores for ChatGPT consistently ranged from 8 to 9 over the 19 scenarios while the median scores for human responses had a wider range from 3 to 6 depending on the domain and situation. The median scores for ChatGPT responses were significantly higher than human responses (all *P* values<.001; Tables 3 and 4). In addition to higher domain scores, ChatGPT scores also demonstrated consistently narrower interquartile ranges (Table 2).

Except for 1 scenario (scenario 11) which was under the domain of clinical quality (standards of health care staff), there were no significant differences between female and male participants’ preferences for either ChatGPT or human responses (Multimedia Appendix 1). More than 76.3% (n=68 for men; n=74 for women) of participants within each sex preferred the ChatGPT responses (Multimedia Appendix 1).

**Figure 2:**
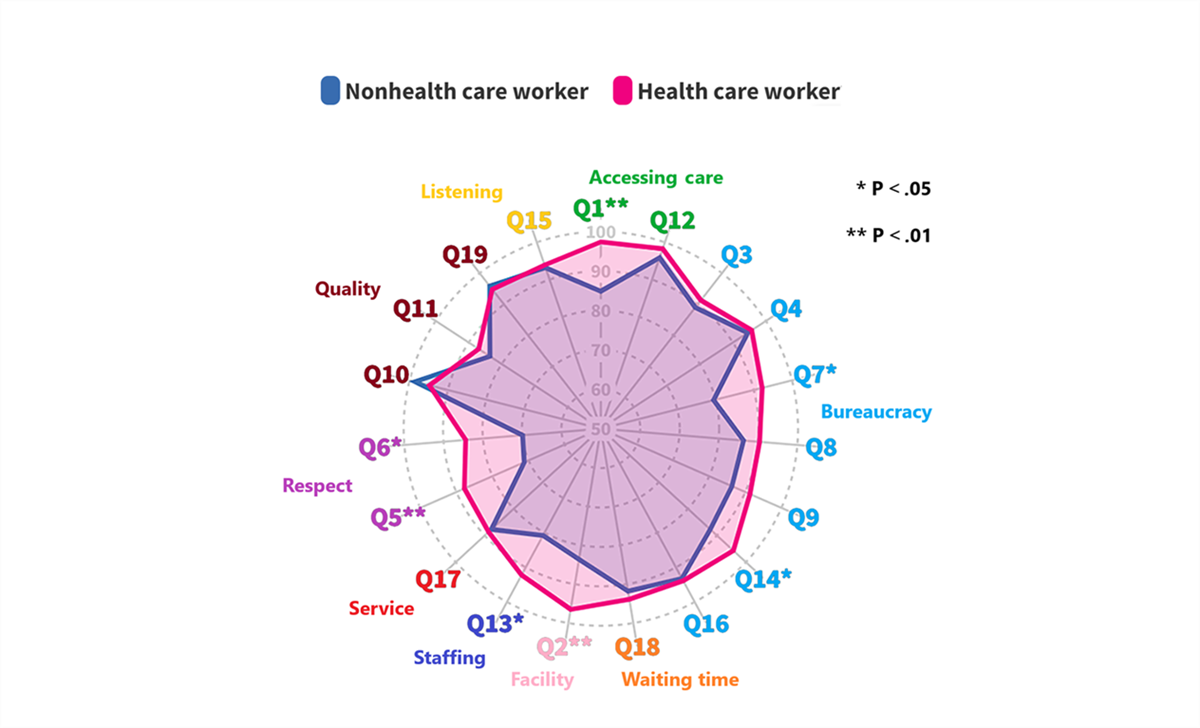
Comparison between health care workers and non–health care workers: proportion of those who preferred ChatGPT responses.

Although majority of the respondents preferred ChatGPT, there were several scenarios where there were higher proportions of non–health care workers preferring human responses, compared to health care workers; and the absolute difference ranged from 8.0% (n=46) to 16.6% (n=49; Figure 2 and Multimedia Appendix 1). These statistically significant differences were seen in scenarios 1 and 14 (management; institutional process; and accessing care), scenario 2 (management; environment; and facility problem), scenarios 5 and 6 (relationship; respect and patient rights; and disrespect), scenario 7 (management; institutional process; and bureaucracy problem), and scenario 13 (management; environment; and staffing problems). In several of these 7 scenarios (1, 2, 6, and 14), health care workers generally graded ChatGPT responses higher in all aspects of appropriateness, empathy, overall quality, and satisfaction, compared to non–health care workers (Multimedia Appendix 2). In contrast, for the human responses, there were no significant differences in the scores for the individual components in almost all the scenarios (Multimedia Appendix 2).

Regression analyses showed that a higher word count was a statistically significant predictor of higher scores in all 4 questionnaire items; every 1-word increment resulted in an increase in scores by 0.015 for appropriateness, 0.019 for empathy, and 0.017 for quality and satisfaction (*P*<.001). On subgroup analysis by authorship, however, this held true only for responses written by PRD staff (*P*<.001), and not for those generated by ChatGPT, which received consistently high scores irrespective of response length (appropriateness *P*=.55; empathy *P*=.33; quality *P*=.60; and satisfaction *P*=.45). The indirect effect of word count appropriateness score (effect=0.68; 95% CI 0.528-0.838), quality score (effect=0.77; 95% CI 0.605-0.929), and satisfaction score (effect=0.71; 95% CI 0.548-0.877) was found to be significant.

One human-written response (case 11) had a word count of 195, comparable to responses written by ChatGPT. When compared against the other human-written responses, it scored higher in all 4 items (*P*<.001). To investigate a comparative difference in scores with ChatGPT responses of similar length, case-control matching for word count was performed with a word count threshold of ±10% for ChatGPT responses. Six of the ChatGPT responses with word counts ranging from 181 to 212 words were matched. Despite a similar word count, these ChatGPT-generated responses still scored higher in all 4 item scores (*P*<.001) than the human-written response.

**Table 2:** Comparison of questionnaire item scores for responses by ChatGPT versus human.

Questionnaire item	ChatGPT, median (IQR)	Human, median (IQR)	*P* value
Appropriateness and completeness	8 (7-9)	5 (3-7)	<.001
Empathy	8 (7-9)	4 (3-6)	<.001
Quality	8 (7-9)	5 (3-6)	<.001
Satisfaction	8 (7-9)	5 (3-6)	<.001

**Table 3:** Results related to management problems (domains)^a^.

Category (subcategory) and question, and response quality				ChatGPT		Human
**Institutional process (accessing care)**						
	**Question 1, median (IQR)**					
		Appropriateness	8 (7-9)		5 (3-6.5)	
		Empathy	7 (6-8.5)		3 (1-5)	
		Overall quality	8 (7-9)		4 (2-5)	
		Satisfaction	8 (7-9)		3 (2-6)	
	Preferred response to question 1, n (%)			174 (92.6)		14 (7.5)
	**Question 12, median (IQR)**					
		Appropriateness	8 (7-9)		3 (2-6)	
		Empathy	8 (7-9)		3 (1-5)	
		Overall quality	8 (7-9)		3 (2-5)	
		Satisfaction	8 (7-9)		3 (2-5)	
	Preferred response to question 12, n (%)			183 (97.3)		5 (2.7)
**Institutional process (bureaucracy problem)**						
	**Question 3, median (IQR)**					
		Appropriateness	8 (7-9)		5 (3-6)	
		Empathy	8 (7-9)		4 (2-5)	
		Overall quality	8 (7-9)		4 (3-6)	
		Satisfaction	8 (7-9)		4 (2-6)	
	Preferred response to question 3, n (%)			170 (90.4)		18 (9.6)
	**Question 4, median (IQR)**					
		Appropriateness	8 (7-9)		5 (3-6)	
		Empathy	8 (7-9)		4 (2-6)	
		Overall quality	8 (7-9)		5 (3-6)	
		Satisfaction	8 (7-9)		5 (3-6)	
	Preferred response to question 4, n (%)			179 (95.2)		9 (4.8)
	**Question 7, median (IQR)**					
		Appropriateness	8 (7-9)		6 (5-8)	
		Empathy	8 (7-8)		5 (3-6)	
		Overall quality	8 (7-9)		5 (4-7)	
		Satisfaction	8 (7-8)		5 (3-7)	
	Preferred response question 7, n (%)			164 (87.2)		24 (12.8)
	**Question 8, median (IQR)**					
		Appropriateness	9 (8-10)		6 (4-8)	
		Empathy	8.5 (8-9)		4 (2-6)	
		Overall quality	9 (8-10)		4 (2-6)	
		Satisfaction	9 (8-10)		5 (3.5-7)	
	Preferred response question 8, n (%)			167 (88.8)		21 (11.2)
	**Question 9, median (IQR)**					
		Appropriateness	8 (8-9)		6 (4-7)	
		Empathy	8 (8-9)		5 (3-6)	
		Overall quality	8 (8-9)		5 (3-7)	
		Satisfaction	8 (8-9)		5 (3-7)	
	Preferred response to question 9, n (%)			168 (89.4)		20 (10.6)
	**Question 14, median (IQR)**					
		Appropriateness	8 (7-9)		5 (3-6)	
		Empathy	8 (7-9)		4 (2-5)	
		Overall quality	8 (7-8)		4 (3-6)	
		Satisfaction	8 (7-8)		4 (2-6)	
	Preferred response to question 14, n (%)			174 (92.3)		14 (7.5)
	**Question 16, median (IQR)**					
		Appropriateness	8 (7-9)		5 (3-6)	
		Empathy	8 (7-9)		4 (3-6)	
		Overall quality	8 (7-9)		5 (3-6)	
		Satisfaction	8 (7-9)		4 (3-6)	
	Preferred response to question 16, n (%)			176 (93.6)		12 (6.4)
**Institutional processes (waiting times)**						
	**Question 18, median (IQR)**					
		Appropriateness	8 (7-9)		5 (3-7)	
		Empathy	8 (7-9)		4 (3-6)	
		Overall quality	8 (7-9)		5 (3-6)	
		Satisfaction	8 (7-9)		5 (3-6)	
	Preferred response to question 18, n (%)			175 (93.1)		13 (6.9)
**Environment (facility problem)**						
	**Question 2, median (IQR)**					
		Appropriateness	8 (7-9)		5 (3-6)	
		Empathy	8 (7-9)		4 (2-6)	
		Overall quality	8 (7-9)		5 (3-6)	
		Satisfaction	8 (7-9)		4 (2-6)	
	Preferred response to question 2, n (%)			172 (91.5)		16 (8.5)
**Environment (staffing problems)**						
	**Question 13, median (IQR)**					
		Appropriateness	8 (7-9)		6 (4-7)	
		Empathy	8 (7-9)		5 (4-7)	
		Overall quality	8 (7-9)		6 (4-7)	
		Satisfaction	8 (7-9)		6 (4-7)	
	Preferred response to question 13, n (%)			165 (87.8)		23 (12.2)
**Environment (service problems)**						
	**Question 17, median (IQR)**					
		Appropriateness	8 (7-9)		5 (3-7)	
		Empathy	8 (7-9)		5 (3-6)	
		Overall quality	8 (7-9)		5 (3-6)	
		Satisfaction	8 (7-9)		5 (3-6)	
	Preferred response to question 17, n (%)			166 (88.3)		22 (11.7)

^a^All *P* values for median (IQR)<.001.

**Table 4:** Results related to clinical and relationship problems (domains)^a^.

Category (subcategory), question, and response quality				ChatGPT		Human
**Respect and patient rights (disrespect)**						
	**Question 5, median (IQR)**					
		Appropriateness	8 (7-9)		7 (5-8)	
		Empathy	8 (7-9)		6 (5-7)	
		Overall quality	8 (7-9)		6 (5-7.5)	
		Satisfaction	8 (7-9)		6 (5-7)	
	Preferred response to question 5, n (%)		153 (81.4)		35 (18.6)	
	**Question 6, median (IQR)**					
		Appropriateness	8 (7-9)		6 (5-8)	
		Empathy	8 (7-9)		6 (4-7)	
		Overall quality	8 (7-9)		6 (5-7)	
		Satisfaction	8 (7-9)		6 (4-7)	
	Preferred response to question 6, n (%)		148 (78.7)		40 (21.3)	
**Quality (clinical standards of health care staff)**						
	**Question 10, median (IQR)**					
		Appropriateness	8 (7-9)		3 (2-5)	
		Empathy	8 (7-9)		3 (1-4.5)	
		Overall quality	8 (7-9)		3 (2-5)	
		Satisfaction	8 (7-9)		3 (1-4)	
	Preferred response to question 10, n (%)		181 (96.3)		7 (3.7)	
	**Question 11, median (IQR)**					
		Appropriateness	8 (7-9)		6 (5-8)	
		Empathy	8 (7-9)		6 (4.5-7)	
		Overall quality	8 (7-9)		6 (5-7)	
		Satisfaction	8 (7-9)		6 (5-7)	
	Preferred response to question 11, n (%)		161 (85.6)		27 (14.4)	
	**Question 19, median (IQR)**					
		Appropriateness	8 (7-9)		4 (2-6)	
		Empathy	8 (7-9)		3 (2-5)	
		Overall quality	8 (7-9)		4 (2-5)	
		Satisfaction	8 (7-8)		3 (2-5)	
	Preferred response to question 19, n (%)		179 (95.2)		9 (4.8)	
**Listening (disregard information from patients)**						
	**Question 15, median (IQR)**					
		Appropriateness	8 (7-9)		5 (3-6)	
		Empathy	8 (7-9)		4 (3-6)	
		Overall quality	8 (7-9)		5 (3-6)	
		Satisfaction	8 (7-9)		4.5 (3-6)	
	Preferred response to question 15, n (%)		176 (93.6)		12 (6.4)	

^a^All *P* values for medians (interquartile range) <.001.

## Discussion

### Principal Findings

The release of ChatGPT and subsequent wave of interest in the use of LLMs in transforming health care has seen multiple hypotheses on the potential impact of their use in patient communications [20,21]. Recent studies have examined the readability and clinical accuracy of LLMs in response to patient or physician queries [22-24], but the evaluation of LLM output against current standard of care, that is, human responses, has yet to be tested rigorously under any form of trial conditions. Ayers et al [12] demonstrated that GPT-3.5 responses to patient questions on an internet forum were assessed to be of higher quality and empathy than verified physician responses, but these were evaluated by a small group of 3 physicians. To our knowledge, our single-blinded study comprising a sizeable group of 188 evaluators across 19 communication scenarios represents the first evidence of the effectiveness of LLMs in patient complaint resolution. GPT-4 demonstrated superiority in response appropriateness, empathy, quality, and overall satisfaction when compared against actual human responses to patient complaints received in an academic medical center. These findings lend credence to the enthusiasm surrounding LLM use in health care settings.

Post hoc, we hypothesized that the reasons for ChatGPT’s superiority could potentially be mediated by the length of responses as measured by word count. Lengthier responses tend to be ranked higher, but our subgroup analysis showed that while scores for human-written responses increased as the word count increased, ChatGPT’s scores did not. These results suggest that a “minimum length” exists for a satisfactory and high-quality response to a patient complaint, with diminishing returns beyond this count. Language models, which can be prompted to generate longer replies or replies of specific length with no additional effort to the user, hold an advantage in this arena. Despite the mediating effect of word count on item scores, ChatGPT still outperformed human-written responses when case-control matched for word count. This suggests an inherent superiority in the quality or writing of ChatGPT’s responses, which could be explored further with a qualitative methodology.

Resolution of patient complaints is a time-intensive endeavor [3] and places additional burdens on hospital staff who must address them. Administrative tasks have been cited as sources of physician burnout [25] and frivolous complaints may distract providers from their primary focus of patient care. The impact of professional complaints against physicians on well-being and mental health are also significant [26]. Evidence from this study suggests that integration of automated replies generated by language models has the potential to alleviate this burden on health care staff, allowing them to dedicate energy and time to other duties.

Health care workers and non–health care workers generally view complaints differently; health care workers often view patients’ complaints as negative, with disregard and ingratitude toward the care that they provide [27], rather than a source of information for further improvements in quality of care. As such, we evaluated if there would be differences in ratings among the 4 domains and preferences between health care and non–health care workers. Interestingly, health care providers tend to rate ChatGPT responses higher than non–health care providers and a greater percentage of non–health care providers preferred human responses compared to health care workers. This could be due to underlying perceptions of the health care providers, who have rooted concepts of what “gold standard” replies should comprise. On the contrary, what patients (who are non–health care professionals) prefer may differ from these perceived standards.

Since there are several studies which find that women demonstrate greater self-reported [28-30] and observed [31] levels of empathy, we conducted a subgroup analysis to evaluate if a higher proportion of women, compared to men, may significantly find ChatGPT responses to be more empathetic and prefer ChatGPT responses to human responses. However, we did not find any differences in preferences between the sex in all but 1 scenario. In this sole scenario which demonstrated a significantly higher proportion of women preferring the ChatGPT response to men (91.8% vs 79.8%; *P*=.02), the individual median scores for empathy and all other domains were not different between the sex. On closer examination, the scenario was the only complaint directly alleging medical negligence, questioning the diagnosis and clinical management of the health care provider. It is possible that there are differences in sex regarding such complaints alleging medical negligence, which are not reflected in the domain scores encompassed by our survey. There are other dimensions to the complaint responses; as shown in our study, most respondents also felt that the ChatGPT responses performed better in terms of quality, completeness, and appropriateness. We suspect that differences in sex in preferences regarding these other domains could have had an additional effect which negated the preferences resulting from differences in sex in empathy, resulting in there being no final difference in overall preference for ChatGPT responses between sexes.

The ChatGPT responses were written with language that was more polite and accommodating compared to those by the PRD. It is likely that this played a part in overall preference scores. Studies have previously shown that perceived physician politeness is associated with increased patient satisfaction [32,33]. Although it is possible that by directing the PRD to write complaint responses in a more polite manner, the preference gap between human and AI responses could potentially be narrowed; we acknowledge the way that ChatGPT can automatically craft responses in an eloquent and polite manner to be one of its intrinsic strengths and characteristic.

### Limitations

There are several limitations in our study. First, while our study evaluated the quality of wholly AI-generated responses, we did not manage to assess how AI could assist rather than completely replace PRD responses. The patient complaints assessed in our study were more straightforward and of limited complexity, without the need for significant fact-finding or interviewing of multiple parties beforehand. The complaints also did not include back-and-forth exchanges, as may occasionally be the case. It is currently unclear how LLMs would perform in the context of more nuanced and complicated complaint scenarios. LLMs could also potentially meet difficulty with maintaining consonance in protracted exchanges. As the use of LLMs in patient-related complaints is still relatively novel, we had intended for straightforward scenarios in our study to evaluate its feasibility.

Second, our study did not manage to evaluate the effectiveness of AI-generated responses based on all the various categories and subcategories of complaint domains [1]. This is because we sought real-life cases, which were available opportunistically, instead of simulated scenarios. It is possible that different domains could yield different results with AI-generated responses. For example, AI-generated responses are hypothetically more likely to perform better with complaints regarding generic health care staff-patient relationships compared to those in the quality of clinical care domain. The latter would require more contextualized knowledge of the clinical case and may also need the input of various data points from the health records, which current LLMs may not be able to achieve. Furthermore, while ChatGPT did not generate impractical complaint resolution measures, this remains an area of concern as the base model does not have domain-specific knowledge, such as hospital policies or standard operating procedures which may vary between health care institutions. Human oversight and verification of LLM output would still be essential in the complaints handling process. Future research avenues include the optimizing of LLM output through comparison of different base models, hyperparameter tuning, and meta-prompting strategies. The development and use of retrieval-augmented generation models, which have been grounded in external data sources from which they may reference, may also overcome some of the limitations related to contextualized and domain-specific knowledge deficits in base models.

Third, despite prompts to limit the word count, the ChatGPT responses tended to be longer with more eloquent prose compared to PRD responses that were universally shorter and occasionally curter. This may have led to failure of blinding, as the distinctly different styles of responses could potentially have helped respondents to identify the ChatGPT response. However, respondents were prompted to select replies that they personally preferred, rather than what they thought were ChatGPT responses.

Fourth, we did not collect other demographics such as education level or socioeconomic status, which may potentially affect personal preferences and thus the results of the survey. It is unclear if different socioeconomic levels may affect an individual’s preference for the more eloquent responses of ChatGPT.

Finally, although we evaluated responses to complaints that came from the general public, and hence a variety of patient backgrounds, a majority of our respondents were health care workers. It is possible that health care workers were more motivated to participate in our survey as it is a subject that is directly relevant to their work, and hence resulted in proportionately more of them responding. We feel that the views of health care workers are still relevant as they are part of the regular workflow in responding to patient complaints—be it drafting or vetting complaint responses. We also managed to compare the differences in preferences between health care and non–health care workers in our discussion.

### Conclusions

Responding to patient complaints consumes a significant number of resources that could otherwise have been used to further patient care. We have shown that LLM-generated responses were universally preferred to the original human responses and thus provide a viable aid to hospital PRDs, potentially leading to cost savings. Future research may be done from the health care system’s point of view regarding the degree of improvement that AI-generated responses can bring in terms of time savings, cost-effectiveness, response quality, and stress reduction.

## Appendix

Multimedia Appendix 1 Table S1. Proportion of participants who preferred ChatGPT responses by subgroup.

Multimedia Appendix 2 Table S2. Scores for individual qualities comparing non-health care and health care workers.

## Abbreviations

AI: artificial intelligence
LLM: large language model
PRD: patient relations department
