# Supplementary material for: Performance of Large Language Models in Patient Complaint Resolution: Web-Based Cross-Sectional Survey
Source: J Med Internet Res. 2024 Aug 9;26:e56413. doi: 10.2196/56413 (PMC11344182; doi:10.2196/56413)
Supplement: Multimedia Appendix 2 [file jmir_v26i1e56413_app2.docx]

**Table S2.** Scores for individual qualities comparing non-health care and health care workers.

| **Domain; category; subcategory** | **Qn** | **Gender, n (%)** | | ***P* value** | **Occupation, n (%)** | | ***P* value** |
| --- | --- | --- | --- | --- | --- | --- | --- |
|  |  | **Male**  **(n = 89)** | **Female**  **(n = 97)** |  | **Non-healthcare worker (n = 73)** | **Healthcare worker**  **(n = 115)** |  |
| **Management; institutional process; accessing care** | 1 | 83 (93.3) | 90 (92.8) | 0.899 | 62 (84.9) | 112 (97.4) | 0.003^a^ |
|  | 12 | 85 (95.5) | 96 (99.0) | 0.195^a^ | 70 (95.9) | 113 (98.3) | 0.378^a^ |
| **Management; institutional process; bureaucracy problem** | 3 | 81 (91.0) | 87 (89.7) | 0.761 | 65 (89.0) | 105 (91.3) | 0.607 |
|  | 4 | 85 (95.5) | 93 (95.9) | 1.000^a^ | 69 (94.5) | 110 (95.7) | 0.737^a^ |
|  | 7 | 80 (89.9) | 83 (85.6) | 0.371 | 58 (79.5) | 106 (92.2) | 0.011 |
|  | 8 | 82 (92.1) | 83 (85.6) | 0.157 | 63 (86.3) | 104 (90.4) | 0.381 |
|  | 9 | 78 (87.6) | 88 (90.7) | 0.498 | 63 (86.3) | 105 (91.3) | 0.278 |
|  | 14 | 83 (93.3) | 89 (91.8) | 0.697 | 64 (87.7) | 110 (95.7) | 0.042 |
|  | 16 | 86 (96.63) | 88 (90.72) | 0.137 | 68 (93.2) | 108 (93.9) | 0.835 |
| **Management; institutional processes; waiting times** | 18 | 81 (91.0) | 92 (94.9) | 0.306 | 67 (91.8) | 108 (93.9) | 0.574 |
| **Management; environment; facility problem** | 2 | 83 (93.3) | 87 (89.7) | 0.386 | 61 (83.6) | 111 (96.5) | 0.003^a^ |
| **Management; environment; staffing problems** | 13 | 77 (86.5) | 87 (89.7) | 0.503 | 59 (80.8) | 106 (92.2) | 0.021 |
| **Management; environment; service problems** | 17 | 80 (89.9) | 84 (86.6) | 0.488 | 64 (87.7) | 102 (88.7) | 0.831 |
| **Relationship; respect and patient rights; disrespect** | 5 | 76 (85.4) | 76 (78.35) | 0.214 | 52 (71.2) | 101 (87.8) | 0.004 |
|  | 6 | 73 (82.0) | 74 (76.3) | 0.337 | 51 (69.9) | 97 (84.4) | 0.018 |
| **Clinical; quality; clinical standards of healthcare staff** | 10 | 85 (95.5) | 94 (96.9) | 0.711 | 72 (98.6) | 109 (94.8) | 0.251 |
|  | 11 | 71 (79.8) | 89 (91.8) | 0.019 | 61 (83.6) | 100 (87.0) | 0.518 |
|  | 19 | 85 (95.5) | 93 (95.9) | 1.000^a^ | 70 (95.9) | 109 (94.8) | 1.000^a^ |
| **Relationship; listening; disregard information from patients** | 15 | 84 (94.4) | 90 (92.8) | 0.658 | 68 (93.2) | 108 (93.9) | 0.835 |

^a^ Fisher’s exact test.
